# Deep brain stimulation of the subthalamic nucleus restores spatial reversal learning in patients with Parkinson’s disease

**DOI:** 10.1093/braincomms/fcae068

**Published:** 2024-02-27

**Authors:** Isabel Schneider, Robby Schönfeld, Annika Hanert, Sarah Philippen, Inken Tödt, Oliver Granert, Maximilian Mehdorn, Jos Becktepe, Günther Deuschl, Daniela Berg, Steffen Paschen, Thorsten Bartsch

**Affiliations:** Memory Disorders and Plasticity Group, Department of Neurology, University Hospital Schleswig-Holstein, Kiel 24105, Germany; Institute of Psychology, Martin-Luther-University Halle-Wittenberg, Halle 06108, Germany; Memory Disorders and Plasticity Group, Department of Neurology, University Hospital Schleswig-Holstein, Kiel 24105, Germany; Memory Disorders and Plasticity Group, Department of Neurology, University Hospital Schleswig-Holstein, Kiel 24105, Germany; Memory Disorders and Plasticity Group, Department of Neurology, University Hospital Schleswig-Holstein, Kiel 24105, Germany; Memory Disorders and Plasticity Group, Department of Neurology, University Hospital Schleswig-Holstein, Kiel 24105, Germany; Department of Neurosurgery, University Hospital Schleswig-Holstein, Kiel 24105, Germany; Memory Disorders and Plasticity Group, Department of Neurology, University Hospital Schleswig-Holstein, Kiel 24105, Germany; Memory Disorders and Plasticity Group, Department of Neurology, University Hospital Schleswig-Holstein, Kiel 24105, Germany; Memory Disorders and Plasticity Group, Department of Neurology, University Hospital Schleswig-Holstein, Kiel 24105, Germany; Memory Disorders and Plasticity Group, Department of Neurology, University Hospital Schleswig-Holstein, Kiel 24105, Germany; Memory Disorders and Plasticity Group, Department of Neurology, University Hospital Schleswig-Holstein, Kiel 24105, Germany

**Keywords:** spatial learning, reversal learning, deep brain stimulation, Parkinson’s disease, subthalamic nucleus

## Abstract

Spatial learning and navigation are supported by distinct memory systems in the human brain such as the hippocampus-based navigational system and the striatum-cortex-based system involved in motor sequence, habit and reversal learning. Here, we studied the role of subthalamic circuits in hippocampus-associated spatial memory and striatal-associated spatial reversal learning formation in patients with Parkinson’s disease, who underwent a deep brain stimulation of the subthalamic nucleus. Deep brain stimulation patients (Parkinson’s disease-subthalamic nucleus: *n* = 26) and healthy subjects (*n* = 15) were tested in a novel experimental spatial memory task based on the Morris water maze that assesses both hippocampal place memory as well as spatial reversal learning. All subjects were trained to navigate to a distinct spatial location hidden within the virtual environment during 16 learning trials in a subthalamic nucleus Stim-On condition. Patients were then randomized into two groups with either a deep brain stimulation On or Off condition. Four hours later, subjects were retested in a delayed recall and reversal learning condition. The reversal learning was realized with a new hidden location that should be memorized during six consecutive trials. The performance was measured by means of an index indicating the improvement during the reversal learning. In the delayed recall condition, neither patients, healthy subjects nor the deep brain stimulation On- versus Off groups showed a difference in place memory performance of the former trained location. In the reversal learning condition, healthy subjects (reversal index 2.0) and patients in the deep brain stimulation On condition (reversal index 1.6) showed a significant improvement. However, patients in the deep brain stimulation Off condition (reversal index 1.1) performed significantly worse and did not improve. There were no differences between all groups in a final visual guided navigation task with a visible target. These results suggest that deep brain stimulation of subthalamic nucleus restores spatial reversal learning in a virtual navigation task in patients with Parkinson’s disease and gives insight into the neuromodulation effects on cognition of subthalamic circuits in Parkinson’s disease.

## Teaser

Dopamine depletion in basal ganglia disorders cause executive dysfunction. Schneider *et al*. report that deep brain stimulation of the subthalamic nucleus in Parkinson’s disease re-established cognitive flexibility in a virtual navigation task. The authors highlight the benefit of brain stimulation on navigation abilities.

## Graphical Abstract

**Graphical Abstract:**
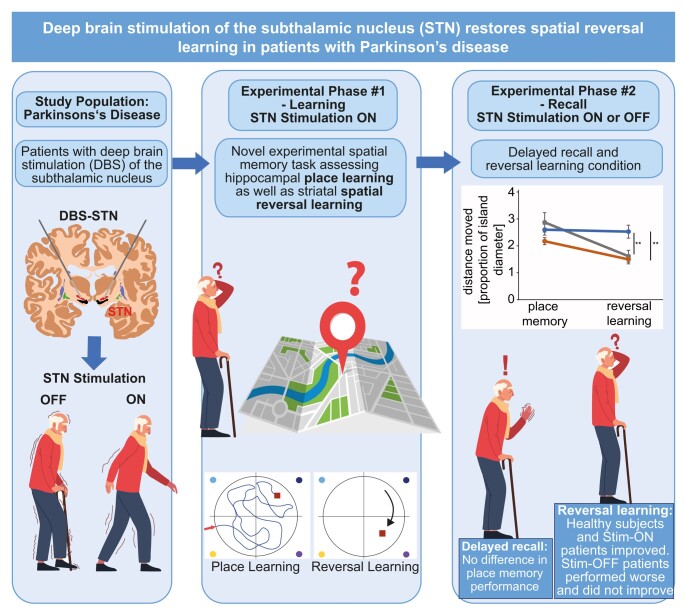

## Introduction

Parkinson’s disease is a progressive chronic neurological disease of the basal ganglia that is associated primarily with motor impairments, including bradykinesia, muscle stiffness, postural instability and rest tremor. But also behavioural, cognitive and vegetative symptoms are major factors in reduced quality of life in the course of the disease and frequently complicate treatment.^1,2^ A multitude of cognitive deficits in Parkinson’s disease has been characterized highlighting an impairment of cognitive flexibility, in which an executive dysfunction results in an inadequate adaptability of strategies or rules.^3,4^ The loss of dopamine-producing neurons in the substantia nigra in the course of Parkinson’s disease leads to a related loss of dopaminergic axons in the striatum which results in an insufficient activation of direct striatal neurons as well as insufficient inhibition of indirect striatal neurons.^5^ This imbalance results in overactivity of the subthalamic nucleus (STN).^3,6^ The STN is critically involved in decision-making and behavioural control.^7,8^ An important hypothesis suggests a functional division of the STN into sensorimotor (dorsolateral), cognitive (ventromedial) and limbic (orbital and medial) parts with pronounced connectivity to cortical regions.^9^ The STN projects to the striatum, the thalamus, the substantia nigra and the primary motor cortex by a complex control loop of the basal ganglia via the globus pallidus.^10^ The STN plays a central role in basal ganglia networks and its main task is to facilitate inhibitory processes in cortico-striato-thalamo circuits and to provide a global ‘no go’ signal.^2^

Deep brain stimulation (DBS) of the STN has proven to be an evidence-based and established treatment of motor symptoms such as tremor, stiffness and bradykinesia. The efficacy of the procedure in the treatment of motor symptoms is widely accepted, but less clear on non-motor and cognitive symptoms.^11^ Different studies have described behavioural changes after STN-DBS, including adverse effects on global cognitive ability, attention, executive function and memory, and the onset of, for example, mania or depression.^2,12,13^ Despite various affected cognitive processes, the cognitive syndrome is often described as a disorder of executive functions, closely related to cognitive flexibility. Two categories of executive functions are usually considered in patients with Parkinson’s disease. On one hand, attention control, such as planning, working memory and task- or set-switching, and on the other hand, the reward-based control of behaviours and risk management.^1^ One assumption is that both categories of executive functions are mediated by cortico-striato-thalamo circuits balanced by an optimal level of dopaminergic innervation in these circuits.^1,2^ An important and well documented finding is the loss of midbrain dopamine neurons, which are mainly part of the nigrostriatal pathway. Thus, the striatal dopamine balance is reduced by about 50–80% even before the first Parkinson’s disease symptoms appear.^14,15^

Cognitive flexibility describes the adaptability of organisms to constantly changing dynamic environmental conditions and the ability to change patterns of thought and reaction and is also essential for successful navigation and the associated switching between different planning strategies and mental representations.^4,16,17^ Cognitive flexibility is also involved in spatial navigation and one component of spatial navigation that requires a particularly high degree of cognitive flexibility is spatial reversal learning. Even though spatial deficits in the course of Parkinson’s disease have been known for years, it has rarely been the focus of research when it comes to Parkinson’s-related cognitive symptoms. Deficits in spatial navigation were already apparent in early stages of Parkinson’s disease.^18^ This impairment has been associated to a progressing dopaminergic deficit.^19^

Spatial navigation is the process in which the subject uses multiple cue sources, such as path integration or landmarks, to navigate the path to a particular target destination. It has been shown that visual processing, attention, executive function and memory contribute to navigation.^11^ Spatial navigation is often associated with hippocampal learning, in the sense of place learning.^20-22^ Spatial place learning is defined as the ability to learn, to encode and to retrieve the location of a given target object irrespective of the starting point of the trajectories. This adaptive and flexible formation of memories of distinct locations in a spatial environment requires a landmark-dependent allocentric navigation. Place learning is strongly dependent on the hippocampus and as such considered a typical hippocampus-dependent function. Besides, spatial navigation also includes striatal functions.^23,24^ Thus, spatial deficits are not only developing after lesions to the hippocampus, but also to cortical and subcortical structures including the dorsal striatum.^25^ Components of spatial navigation thus seem to be the result of an adaptive complex interaction of hippocampus and basal ganglia.^23,24,26-28^

Here, we investigated the influence of STN-DBS on hippocampus-associated spatial navigation and studied decision processes in a spatial context by means of a spatial reversal learning task. We used a modified version of a virtual water maze task. The strength of this task lies in the fact that both place learning and reversal learning can be tested.

STN-DBS was switched off in half of the patients after spatial learning in the virtual water maze task. Since untreated Parkinson’s disease is associated with loss of cognitive flexibility and deficits in executive functions, we thus hypothesized emerging deficits with spatial reversal learning in STN-Off patients whereas a stable or restored performance was assumed with active STN-DBS.

## Materials and methods

### Participants

Twenty-six patients with Parkinson’s disease that received DBS of the STN and 15 healthy controls were prospectively recruited from the Kiel Center—DBS in Movement Disorders Program. Inclusion criteria for the patient group were the clinical diagnosis of idiopathic Parkinson’s disease^29^ for at least 5 years, an age between 47 and 78 years, and that the DBS surgery took place less than 12 months ago. DBS surgery was performed in a standardized procedure as described in Deuschl *et al*.^30^ and Witt *et al*.^31^ In all patients, the DBS was induced using the model 3389 DBS (Medtronic), connected to a pulse generator (Kinetra, Medtronic). Exclusion criteria were a major psychiatric illness, operative contraindications and dementia (Mattis Dementia Rating Scale, sum score ≤ 130^32^). Severity of Parkinson’s disease was assessed using the dose of Levodopa-equivalent medication (mg/day), the total score of the Unified Parkinson Disease Rating Scale III (UPDRS III^33^) before and after turning off the DBS, and the duration of illness. All participants were tested in the morning for general psychological assessment and the learning condition of spatial navigation task, and the delayed recall was consistently assessed in the afternoon after a 4 h delay. Medication was left unchanged during the whole test session with an adequate delay of 1 h between intake and testing. Twenty-four patients were on levodopa or other Parkinson’s medication such as dopamine agonists (14 patients), N-methyl-D-aspartate receptor antagonist (8 patients), catechol-o-methyl-transferase inhibitors (12 patients), or monoamine oxidase inhibitors (6 patients). The study was approved by the local ethics committee, and all participants gave informed consent before inclusion.

### Spatial navigation task

**Figure 1:**
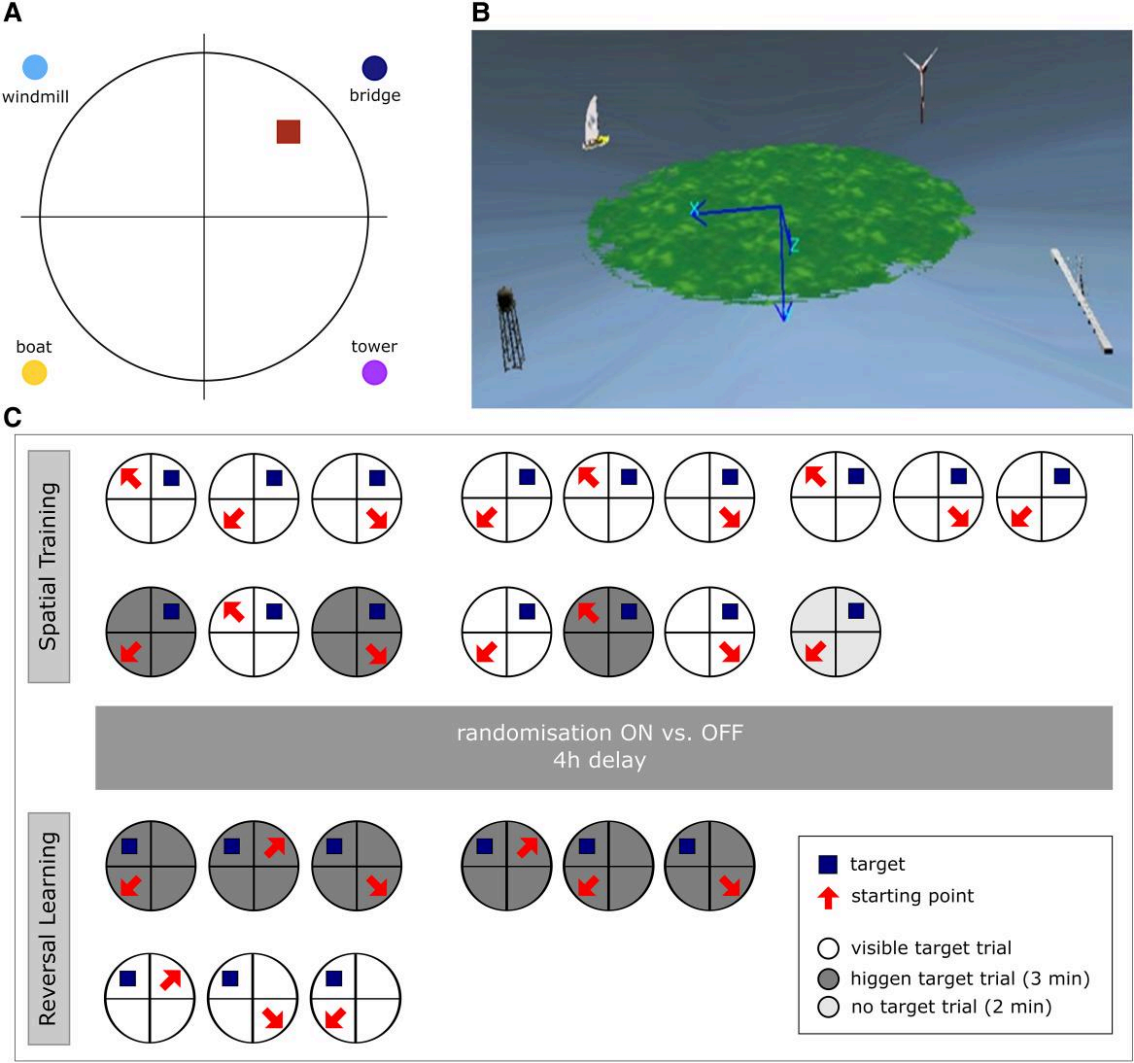
**Sketch of the vWMT.** (**A**) Top view of the island with quadrants, landmarks and location of the treasure chest (target). (**B**) Birdseye view of the virtual island. (**C**) Test protocol indicating the two phases of the vWMT procedure. Spatial learning and reversal learning with the square indicating the target locating and the arrow indicating the starting position. Note that half of the DBS patient group had the STN stimulation switched Off after the 4 h delay. vWMT, virtual water maze task; DBS, deep brain stimulation; STN, subthalamic nucleus; min, minutes; h, hours.

Patients (*n* = 26) receiving bilateral DBS were tested on the spatial navigation task. During the spatial learning trials (Fig. 1), all patients had their STN-DBS On. Directly after learning, patients were randomized into two groups, either with DBS On (*n* = 13, On–On group) or DBS Off (*n* = 13, On–Off group) such that the recall and spatial reversal learning were performed 4 h after the randomized change of the patients’ DBS condition.

We used a spatial navigation task based on the Morris water maze task, a behavioural test of hippocampus-dependent learning and memory in rodents.^34^ The Morris Water Maze is considered as one of the most important behavioural tasks for the assessment of hippocampus-associated spatial learning and place navigation.^21^ We used a computer-based virtual version of the paradigm virtual water maze task which we evaluated in patients with hippocampal dysfunction.^35^ The maze is conceptualized as a virtual island on which a treasure chest had to be found as an equivalent to the platform of the original water task. Four orthogonal landmarks (a yacht, wind turbine, bridge and water tower) were located off-shore adjacent to the coastline. The island was virtually divided into four quadrants, with the target in the quadrant close to the bridge (Fig. 1A and B).

The task has been run into two phases: (i) spatial learning, and (ii) recall and (iii) spatial reversal learning.^36,37^

The virtual water maze task used in this study was modified towards a spatial memory decision-making process. As the aim of the paradigm was to evaluate the effect of the neuromodulatory influences of STN stimulation on mnemonic spatial decision-making, a spatial reversal learning task was incorporated into the paradigm. Reversal learning is defined as a shifting between different stimulus–reward associations.^38^ In the vWMM, it is conceptualized in distinct reversal learning trials during which the patient had to learn the new location of the target object as this was relocated to a new location (Fig. 1C). In our paradigm, reversal learning was implemented with a new hidden location that should be stored during six ongoing trials in terms of moving the treasure chest to another location in another quadrant. The performance was measured by means of an index showing the improvement during the reversal learning. As a prerequisite of reversal learning, successful hippocampus-dependent encoding and consolidation was a critical component in evaluating spatial reversal learning.

### Procedure

The task began with a pre-task training unit in joystick use, motor training, as well as training in the immersive nature of the VR (virtual reality) task. No off-shore cues were available and participants had to navigate to seven visible treasure boxes during this trial. No time limit was given and no measures were obtained from the joystick training. Participants performed the navigation task at a 24 in. LED monitor. For moving over the island, a standard video game joystick was used, which was calibrated before beginning the data acquisition.

#### Spatial learning

The spatial learning phase included 16 trials (Fig. 1C). During each trial, participants were placed block-randomized to one of three start positions of the off-shore quadrants (opposite, left and right adjacent of the target quadrant) and given 3 min to navigate to the target. The presentation of the box was done in four different ways: visible (visible target trials), hidden in a hollow, slightly elevated above ground level of the island (hidden target trials) and not present at all (no target trials).

From their start positions, participants had to navigate to the target. When they reached their destination, the treasure chest, a positive acoustic signal sounds and a congratulations screen appeared. This function served as a reward. If the box had not been successfully located within 2 min, the target box was elevated above the ground surface of the island and could now be located and navigated by visual guidance. So, whenever the box was reached, participants were given an on-screen confirmation and asked to look around and attend to the environmental features and landmarks. After then, they could proceed to the next trial by pressing a key. Pilot experiments had shown that patients benefit form forward movements only. Hence, to compensate for individual motor capabilities, only a forward movement was possible but not a backward movement.

The first 9 consecutive trials were visible target trials, and trials 10, 12 and 14 were hidden target trials. The target was made alternating visible and hidden during trials 10–15 serving as a condensed training procedure to quickly acquire a hippocampus-dependent place memory.

Trial 16 was administered immediately after the spatial learning and was designed as a no-target probe trial, in which the treasure box was completely removed. Piloting and previous studies showed that subjects who reported stable mental representation of the acquired target showed a strong and reliable preference for the target quadrant in this state for at least 1–2 min, so this trial duration was limited to 2 min.^21^ Effective and successful encoding of hippocampus-dependent spatial memory and place learning of participants was ensured by incorporating (i) alternating hiding and visualization of the chest, (ii) an immediate recall (no-target trials) and (iii) a delayed recall condition incorporated into the first trial of the reversal procedure (see next section).

#### Reversal learning

After a delay of 4 h to allow for consolidation of spatial memory and stabilizing the effects of switching off the DBS, successful place learning was tested using the first 2 min of the first reversal trial as a delayed free recall task. In total, six reversal learning trials were performed with the treasure box located in a new position in a different quadrant (new target position). Importantly, the fact that the target was now in a new location was not explicitly indicated to the participants. So that the people themselves had to realize that the old target position was no longer existing for them and they had to search for the chest in a new position. The landmarks remained at identical locations. The new target position was retained for all reversal trails to induce a stable bias and drive for spatial reversal learning which meant that only through successful spatial reversal learning an efficient navigation and relearning to the new target location (reversal learning condition) was possible. Each trial was again limited to 3 min. Identical to the spatial learning condition, if the target could not be reached within the time limit of 2 min, its position was revealed by raising it slightly above the ground level of the island. During trial 1–6, the box was hidden in a hollow, from trial 7, the box was visible for the last three trials (visible target trials), serving again as a motor control condition (Fig. 1C). Participants were instructed to behave the same as before in the spatial training procedure. This motor control condition is implemented to distinguish cognitive from motor symptoms in the DBS Off group.

### Analysis

Behavioural parameters measured during the spatial learning sessions were (i) total distance moved, measured the trajectory from start location to target in proportion to the pool diameter and (ii) latency, the time taken to locate the target, measured in seconds (called ‘escape latency’ in animal experiments). Both measures were averaged over three runs and combined into blocks (block of trials 1–3, trials 4–6 and trials 7–9). These averages per block describe the performance of the visible target trials. Further variables were computed as means on trials 10–15 and measure performance of the alternating hidden and visible target training. In trial 16, where the treasure box was completely removed and served as immediate recall of place learning, we measured relative dwell times, specified as the pro rata time per quadrant (time within a specific quadrant relative to the total duration of the probe trial).

The first reversal trial was also analysed as a probe trial (delayed recall of place learning; see above). Relative dwell times were computed from the first 120 s of this trial in the same way as in trial 16.

Spatial reversal learning was analysed during the trials using also the total distance moved measure. For this purpose, trials were again grouped into blocks (block 1: reversal trials 1–3 and block 2: reversal trials 4–6).

The ratios of the dependent measures of these two blocks (block 1/block 2) were termed ‘savings’ indicating an improvement or deterioration during reversal learning, where values above 1.0 indicate improvements. Trials 7–9 with visible targets were used to assess the visuo-motor control via the total distance moved measure.^36^

In addition, the performance of the patients with successful hippocampal place learning was classified by means of *z*-score-based internal criteria. Criteria for ‘Learner’ were (i) a total distance moved during the alternating learning block less than a standard deviation above the average of the healthy controls (*c*_learn_ ≤ *M*_con_ + SD_con_ = 1.74 pool diameter), (ii) a relative dwell time in the target quadrant during immediate recall longer than a standard deviation below the mean of the controls (*c*_im_ ≥ *M*_con_ − SD_con_ = 0.47) and (iii) a relative dwell time longer than half a standard deviation below the mean of the controls during delayed recall (*c*_de_ ≥ *M*_con_ − 0.5 SD_con_ = 0.25). A ‘Reversal Learner’ criterion was administered to classify patients with successful spatial reversal learning. For this, the saving index had to be at least one half standard deviation below the average savings of the controls (*c*_sav_ ≥ *M*_con_ + SD_con_ = 1.51).

Patients who are considered ‘Learners’ and ‘Reversal Learners’ are classified as ‘real-Reversal Learner’, indicating that they successfully showed hippocampal place learning as well as successful reversal learning.

This classification was used to assess possible associations of the STN electrode locations with the performance in the virtual water maze task in the electrode location analysis.

The latency measure was no longer used to assess the performance in the delayed recall condition, since half of the patients had their stimulators switched off and their Parkinson’s symptoms were ‘untreated’ resulting in a hypokinetic state with a much slower motor speed.

### Neuropsychological testing

The neuropsychological assessment included the established Test Battery of Attentional Performance (TAP^39^) to test a number of attention functions by means of a set of reaction time tasks including subtest of arousal (alertness task), cognitive flexibility (dual task) and impulsivity (go/nogo task). The TAP was also carried out twice, before and after the 4 h delay. Participants also completed the trail-making-test A/B,^40^ a standard measure of visual scanning, processing speed and cognitive flexibility, the Regensburg word fluency test^41^ that assesses executive functioning and the Digit Span (forward and backward) according to the Wechsler Adult Intelligence Scale^42^ for the assessment of working memory. The premorbid intelligence was estimated via the level of crystallized intelligence and was measured by means of a German version of the national adult reading test.^43^

The Geriatric Depression Screening Scale^44^ gave an impression of dysphoric mood in our patients and controls. The handedness was elevated by means of the Edinburgh Handedness Inventory.^45^

Neurological examination included the UPDRS III^33^ which was initially performed by all patients. The patients of the DBS On–Off group performed the test again after switching off the stimulator to assess the magnitude of the stimulation effect on motor symptoms.

### Magnetic resonance image acquisition and data analysis

To localize the STN and to verify the correct contact positions of the DBS leads in all patients, T1-weighted structural MR imaging was performed on a Philips Medical Systems, Intera Achieva at 1.5 T. All patients were scanned before (pre-acquisition) and 1–2 days after DBS surgery (post-acquisition). Pre-images were acquired with the following MR parameters: repetition time = 10.8 ms, echo time 2 ms, flip angle = 30°, field of view = 250 mm × 250 mm, 256 × 256 matrix and 160 slices of 1 mm thickness. Post-images were acquired with slightly different parameters to account for the DBS stimulator safety requirements. The post-MR images were acquired with the parameters: repetition time = 25 ms, echo time 4.6 ms, flip angle = 20°, field of view = 229 mm × 229 mm, 256 × 256 matrix and 160 slices of 0.8 mm thickness. Data of pre- and post-examinations were co-registered and normalized with the SPM (Statistical Parametric Mapping) (‘http://www.fil.ion.ucl.ac.uk/spm/’) toolbox using the co-registration and segmentation function using Matlab 7.7.0 (MathWorks).

Lead midline were determined by manually placing between 5 and 8 control points along the centre of the image artefact of each electrode within the postoperative image. The MR image was then re-sliced along this lead midline to visualize the MRI signal intensities and artefacts along the lead. The four electrode contacts at the tip of each lead cause special artefacts that can be identified in this intensity profile. The final depth of the electrodes was interactively determined by fitting an electrode shape model to this specific MRI artefact along the lead midline.

To compare the electrode positions over all patients, subject-specific active contact positions were normalized to a common (normalized) Montreal Neurological Institute (MNI) coordinate space. The exact spatial coordinate normalization transform to a common MNI template coordinate system was determined from the images before surgery using the SPM segment function (which includes an optimized image normalization with an integrated intensity inhomogeneity correction). Active electrode contact positions were then transferred by image co-registration from the post-images to the pre-image and finally to the MNI coordinate by applying the non-linear normalization transform.

The exact positions of the left and the right active electrode contacts were calculated by referring to the three coordinates of the MNI space, as well as the Euclidean distance to the STN midpoint. The sweet spot of the left (−12.68, −13.53 and −5.38 mm) and the right STN (12.50, −12.72 and −5.38 mm) in MNI space was derived from the atlas by Dembek *et al*.^46^ A more detailed description of the procedure can be found in Volkmann et al.^2^ Additionally, we compared the stimulation voltage and currents between the two patient groups (DBS On–On and DBS On–Off**)** (Table 1).

**Table 1:** Applied stimulation parameters for groups DBS On–On (On) and On–Off (Off)

Coordinates/stim. parameters	DBS On	DBS Off	ANOVA
*Left hemisphere*			
Active electrode X	−10.96 (0.52)	−10.35 (0.45)	*F*(1, 20) = 0.70; *P* = 0.42
Active electrode Y	−14.20 (0.41)	−13.91 (0.44)	*F*(1, 20) = 0.23; *P* = 0.64
Active electrode Z	−7.28 (0.60)	−7.52 (0.81)	*F*(1, 20) = 0.06; *P* = 0.81
Voltages (Volt)	2.53 (0.31)	3.03 (0.21)	*F*(1, 20) = 0.41; *P* = 0.53
Frequency (Hertz)	143.33 (8.56)	164.44 (9.59)	*F*(1, 20) = 0.23; *P* = 0.62
Euclidean distance to the STN	4.33 (0.33)	4.53 (0.43)	*F*(1, 20) = 3.71; *P* = 0.07
*Right hemisphere*			
Active electrode X	12.44 (0.40)	11.58 (0.53)	*F*(1, 20) = 1.79, *P* = 0.20
Active electrode Y	−14.29 (0.51)	−13.64 (0.39)	*F*(1, 20) = 0.91, *P* = 0.35
Active electrode Z	−6.92 (0.70)	−7.86 (0.60)	*F*(1, 20) = 0.96, *P* = 0.34
Voltages (Volt)	2.71 (0.32)	2.90 (0.26)	*F*(1, 20) = 0.01, *P* = 0.91
Frequency (Hertz)	143.33 (8.56)	170.00 (8.66)	*F*(1, 20) = 0.03, *P* = 0.87
Euclidean distance to the STN	3.43 (0.34)	4.69 (0.28)	*F*(1, 20) = 0.61, *P* = 0.45

*Note*. Electrode data were given as means (SEM in parentheses). A single factor ANOVA was used to determine whether there were significant differences in the positions of the left and right activated electrodes and in the stimulation current and voltage between the On–On and On–Off groups (see Fig. 8).

### Statistical analysis

The data analysis was performed with the Statistical Package for the Social Sciences, 17.0. The descriptive analysis expressed the data as group means ± standard error of the means (SEM). Differences in group means were tested by means of ANOVAs with the between-subjects factor control group versus DBS On–On versus DBS On–Off. Shapiro–Wilk test (normality) and Levene statistics (variance homogeneity) were applied to all parameters before statistical testing group differences were tested post hoc with the Tukey multiple comparison method. Non-normally distributed data are evaluated in the following using the non-parametric Kruskal–Wallis test. For this purpose, the post hoc test Dunn–Bonferroni is used to determine which groups differ significantly. If there is no variance homogeneity in the groups, the robust Welch test is used. It is followed by the post hoc Tamhane-T2 test, which is considered a conservative pairwise comparison test based on a *t*-test and is suitable for unequal variances. Correlations between neuropsychological measures and spatial performance (vMWT) were tested by means of Pearson coefficients.

Pairwise deletion was applied to missing data.

The level of significance was set to 0.05. Significant *P*-values are marked with asterisk icons, where ‘*’ labels values *P* ≤ 0.05, ‘**’ *P* ≤ 0.01 and ‘***’ *P* ≤ 0.001.

## Results

The data of all 41 subjects enrolled in this study could be included in the analysis (13 Parkinson’s disease patients with STN-DBS On–On, 13 Parkinson’s disease patients with STN-DBS On–Off and 15 age and gender matched healthy controls). Furthermore, basic characteristics such as handedness, severity of the disease in patients only, as well as neurological and neuropsychological data did not differ significantly between the three groups (Tables 1 and 2). During TAP baseline testing and the spatial learning phase, all Parkinson’s disease patients had their DBS stimulation ‘On’. Directly after this testing phase, patients were randomized and in one half of the patients group, the STN-DBS was switched ‘Off’ (see also ‘Procedure’ in Methods section). The TAP retesting and the spatial reversal learning were performed either in a STN stimulation ‘On’ or ‘Off’ condition (labelled as DBS On–On versus DBS On–Off group in Table 2).

**Table 2:** Baseline characteristics of the sample

Parameter		Controls (*n* = 15)	DBS On (*n* = 13)	DBS Off (*n* = 13)	*P*-values
Age, range in years		62.9, 55–78 (1.0)	60.5, 47–70 (1.7)	63.1, 52–72 (1.7)	0.5
Gender		7:8	9:4	10:3	
Duration of disease (months)			173.4 (22.1)	172.9 (17.2)	0.99
Levodopa-equivalent dose (mg per day)			477.5 (80.6)	350.4 (73.4)	0.26
UPDRS III	Baseline		13.6 (1.9)	14.2 (1.4)	0.80
	Retesting		13.7 (1.8)	30.5 (2.9)	<0.001
EHI		86.7 (8.6)	88.9 (5.1)	81.5 (6.9)	0.2^a^
TMT (total)		69.4 (4.5)	93.8 (15.2)	82.0 (5.3)	0.1^b^
RWT		17.1 (1.3)	15.2 (1.11)	13.8 (1.2)	0.2
Digit Span (total)		6.8 (0.3)	6.7 (0.6)	7.0 (0.5)	0.9
MWT-B		30.7 (0.8)	31.7 (1.5)	31.2 (1.1)	0.8
GDS		1.7 (0.2)	3.5 (0.9)	3.6 (1.0)	0.2^a^
TAP					
Arousal	Baseline	307.4 (33.3)	341.6 (21.4)	328.0 (19.5)	0.6
(Alertness task)	Retesting	293.6 (24.7)	348.3 (23.2)	366.0 (35.3)	0.3
Cognitive flexibility	Baseline	2.7 (0.5)	5.2 (1.4)	4.3 (1.1)	0.2
(dual task)	Retesting	1.3 (0.5)	4.8 (1.6)	4.2 (0.8)	0.01^b^
					Con versus Off:
					0.03^c^
Impulsivity	Baseline	1.0 (0.7)	2.7 (1.2)	2.5 (1.6)	0.6
(Go/nogo task)	Retesting	0.6 (0.2)	0.3 (0.2)	2.3 (1.0)	0.05^a^
					Off versus On:
					0.05^d^

*Note*. Statistic comparisons of group mean differences between controls versus On–On versus On–Off.

Data were given as group means (SEM).

UPDRS, Unified Parkinson’s Disease Rating Scale; EHI, Edinburgh Handedness Inventory; TMT, trail marking test; RWT, Regensburger Wortflüssigkeits test; MWT-B, Mehrfachwahl Wortschatz Intelligenztest; GDS, Geriatric Depression Scale; TAP, Testbatterie zur Aufmerksamkeitsprüfung.

Tests for statistical evaluation: ^a^Kruskal–Wallis H test, ^b^Welch test, ^c^Tamhane-T2 test and ^d^Dunn–Bonferroni test; otherwise ANOVA.

Accordingly, the UPDRS III score differed significantly in the DBS On–Off group in the retest condition. After the patients had their stimulation switched off, the DBS On–Off patients had a significantly higher UPDRS III score than the DBS On–On patients [*F*(1, 24) = 24.24, *P* < 0.001, Cohens’ *d* = 6.9]. The cognitive flexibility (measured by the dual task of the TAP) and the impulsivity (measured by the go/nogo task of the TAP) also differed significantly between the groups after the STN-DBS was switched off. There was a significant group difference in the dual task subtest at the second onset [Welch’s *F*(2, 19.46) = 5.79, *P* = 0.01]. Whereby a post hoc Tamhane-T2 test showed that the patients in the DBS On–On group made significantly fewer omissions (*P* = 0.03) than the patients in the DBS On–Off group, [no of omissions: −2.90, 95% CI (confidence interval) (−5.43, −0.36); Cohen’s *d* = 4.4]. In addition, a Kruskal–Wallis test showed a significant group difference in the go/nogo task (*χ*^2^ = 40.82; *P* = 0.05), whereby the patients with the stimulator switched Off made significantly more errors than the patients whose brain stimulator remained switched On, which a Dunn–Bonferroni test showed (*z* = 2.4, *P* = 0.05, Cohen’s *d* = 2.8).

There were no significant differences in other neurocognitive parameters between patients and the healthy controls (Table 2).

### Spatial encoding

**Figure 2:**
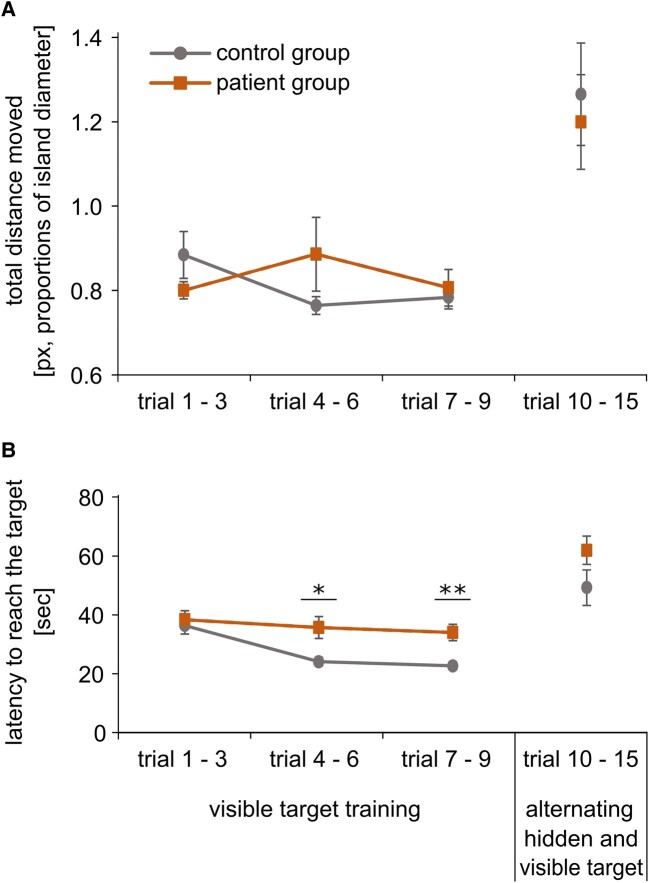
**Spatial learning.** (**A**) Total distance moved during spatial training. A comparison of the control group and the entire patient group, with all the stimulators switched on at that time indicating no significant differences between controls and patients (all *P*’s > 0.05). The diagram shows the mean values of the path length with SEM (px, proportion of island diameter). (**B**) Latency to reach the target. Comparison of the control group and the entire patient group, with all the stimulators switched on at that time with significant higher latencies in the patient group in trials 4–6 [*F*(1, 39) = 5.28, *P* = 0.03] and trials 7–9 [*F*(1, 35.2) = 7.09, *P* = 0.001, ANOVA]. The diagram shows the mean values of the latency in seconds with SEM (Table 3). px, pixel; s, seconds; SEM, standard error of the means (*P ≤ 0.05, **P ≤ 0.01).

The spatial learning was conducted in the patient group and controls. During spatial learning, controls and all patients, regardless of the subsequent stimulation group, showed similar total distances moved (Fig. 2A) and latencies (Fig. 2B) until they reached the chest. An ANOVA showed almost no significant variations between the groups of healthy controls and patients in the alternating hidden and visible target trials 10–15. There were no significant differences between patients and controls in the total distance moved (all *P*’s > 0.05). Only the patients needed significantly longer to reach the target (latencies) than the healthy controls between trials 4–6 [*F*(1, 39) = 5.28, *P* = 0.03] and trials 7–9 [*F*(1, 35.2) = 7.09, *P* = 0.001].

### Spatial recall

**Figure 3:**
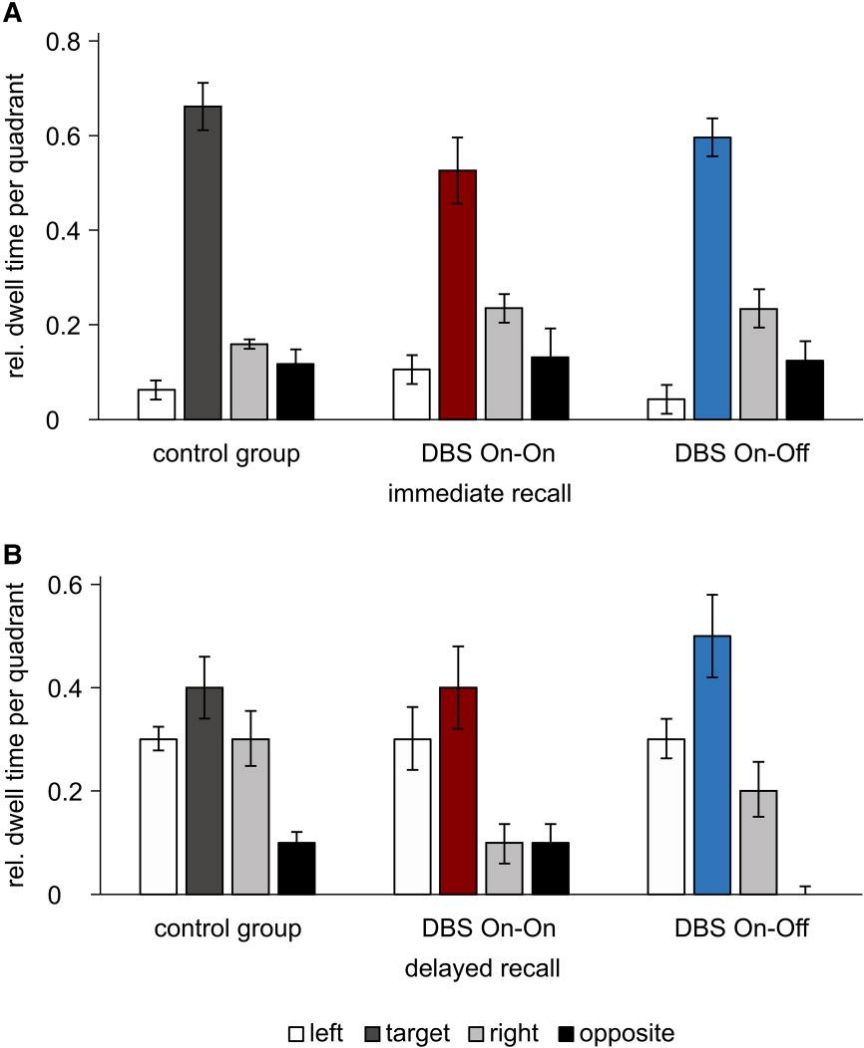
**Relative dwell times.** Group means of relative dwell times in the target quadrant (red bar for DBS On–On and blue bar for DBS On–Off) and the quadrants to the left, right and opposite with SEM (overview in Table 3). (**A**) Immediate recall (trial 16) and (**B**) Delayed recall first trial reversal learning. rel. dwell time, relative dwell time; SEM, standard error of the means.

**Figure 4:**
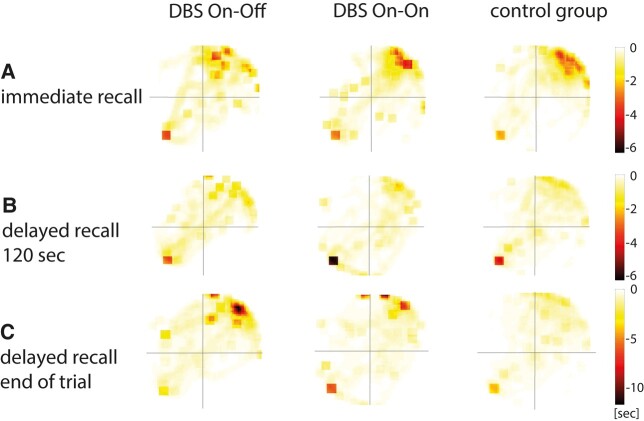
**Heat maps of dwell times.** Increased darkness (red tones) indicates increased average dwell time at the location within the maze area. Square in the downer left quadrant indicates starting position, and upper right quadrant is the target quadrant. (**A**) First 2 min of the no-target probe trial (trial 16). A high dwell time in the target quadrant is visible, marked in red. (**B**) First 2 min of the hidden target probe trial (first reversal). Here, a consistent yellow colouring of the target quadrant is seen in delayed retrieval, indicating successful place memory. (**C**) End of the hidden target probe trial. This shows that the On–Off group still has a high retention rate in the formal target quadrant (Table 3). DBS, deep brain stimulation; sec, seconds.

Hippocampus-mediated place learning performance was measured via relative dwell times during the immediate recall and the delayed recall and was similar in the three groups (Fig. 3). Immediate recall (trial 16—no-target probe) and delayed recall (initial phase of trail 1—reversal learning) showed no significant differences between the three groups with regard to the relative dwell times in all quadrants (target, left, opposite and right). Figure 4 illustrates dwell times as heat maps (Table 3).

**Table 3:** Immediate and delayed recall

Parameter	Controls	DBS On	DBS Off	ANOVA
*Immediate recall*				
Left	0.06 (0.03)	0.11 (0.03)	0.04 (0.07)	*F*(2, 38) = 1.28, *P* = 0.29
Target	0.66 (0.05)	0.53 (0.07)	0.60 (0.04)	*F*(2, 38) = 1.60, *P* = 0.22
Right	0.16 (0.01)	0.24 (0.03)	0.23 (0.04)	*F*(2, 21.27) = 3.99, *P* = 0.03^a^
Opposite	0.12 (0.03)	0.13 (0.06)	0.12 (0.04)	*F*(2, 38) = 0.03, *P* = 0.97
*Delayed recall*				
Left	0.25 (0.03)	0.33 (0.08)	0.30 (0.05)	*F*(2, 21.31) = 0.71, *P* = 0.50
Target	0.38 (0.06)	0.42 (0.08)	0.49 (0.08)	*F*(2, 38) = 0.53, *P* = 0.60
Right	0.28 (0.07)	0.12 (0.05)	0.18 (0.07)	*F*(2, 38) = 1.75, *P* = 0.19
Opposite	0.09 (0.03)	0.14 (0.05)	0.04 (0.02)	*F*(2, 21.17) = 2.67, *P* = 0.09^a^

*Note*. Data were given as means (SEM in parentheses).

ANOVA and ^a^Welch test (Fig. 3).

### Spatial reversal learning

**Figure 5:**
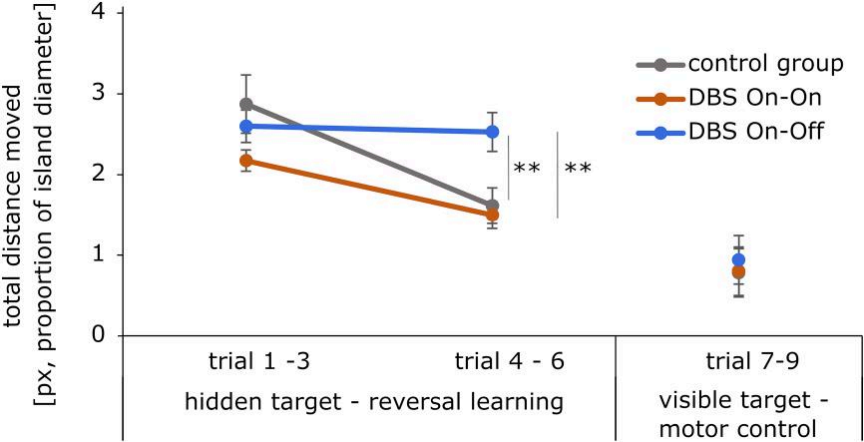
**Spatial reversal learning.** Total distance moved in trials 1–9 of the reversal learning condition. Shown are group means and SEM error bars showing differences between groups in trials 4–6 [*F*(2, 38) = 6.74, *P* < 0.01]. Tukey post hoc analysis revealed a higher reversal learning performance of DBS On–On group compared to DBS On–Off [−1.03, 95% CI (0.27,1.78), *P* < 0.01] as well as DBS On–Off compared to controls [0.91, 95% CI (0.18,1.64), *P* = 0.01, ANOVA]. Trials 7–9 in which visible target served as motor control (Table 4). px, pixel; SEM, standard error of the means; DBS, deep brain stimulation.

In the phase of spatial reversal learning (new target position), patients in the DBS On–Off condition performed significantly worse and did not improve compared to the On–On group as the patients whose DBS was switched off showed significantly longer distance (Fig. 5) until they reached their target in trials 4–6.

The total distance moved in trials 4–6 differed statistically significant between the three groups, *F*(2, 38) = 6.74, *P* < 0.01, where a Tukey post hoc analysis revealed a significant difference (*P* < 0.01) between the groups of DBS On–On and the DBS On–Off [−1.03, 95% CI (0.27,1.78)] and a significant difference (*P* = 0.01) between the group of DBS On–Off group and the controls [0.91, 95% CI (0.18,1.64)] (Table 4).

**Table 4:** Reversal learning

Parameter	Controls	DBS On	DBS Off	ANOVA
*Total distance*				
Trials 1–3	2.87 (0.36)	2.17 (0.13)	2.60 (0.2)	*F*(2, 23.25) = 2.74, *P* = 0.09^a^
Trials 4–6	1.61 (0.22)	1.50 (0.17)	2.53 (0.24)	*F*(2, 38) = 6.74, ***P* <** **0.01**
				On versus Off: *P* < 0.01^b^
				Con versus Off: *P* = 0.01^b^
Savings	2.10 (0.31)	1.64 (0.19)	1.09 (0.09)	*F*(2, 21.54) = 7.31, ***P*** **= 0.004**^a^
				Off versus On: *P* = 0.05^c^
				Off versus Con: *P* = 0.02^c^
*Motor control*				
Trials 7–9	0.78 (0.03)	0.80 (0.07)	0.94 (0.09)	*F*(2, 18.94) = 1.21, *P* = 0.32^a^

*Note*. Data were given as group means (SEM). Significant *P*-values are printed in bold type.

Tests for evaluation were ^a^Welch test, ^b^Tukey post hoc test, ^c^Tamhane-T2 test.

The time to target differed between the groups with respect to all trials (1–3, 4–6 and 7–9). The patients of the DBS On–Off group needed significantly longer in all trials than the healthy controls. They also needed more time to find the target in all trials than the patients of the DBS On–On group, whereby the differences were not significant.

**Figure 6:**
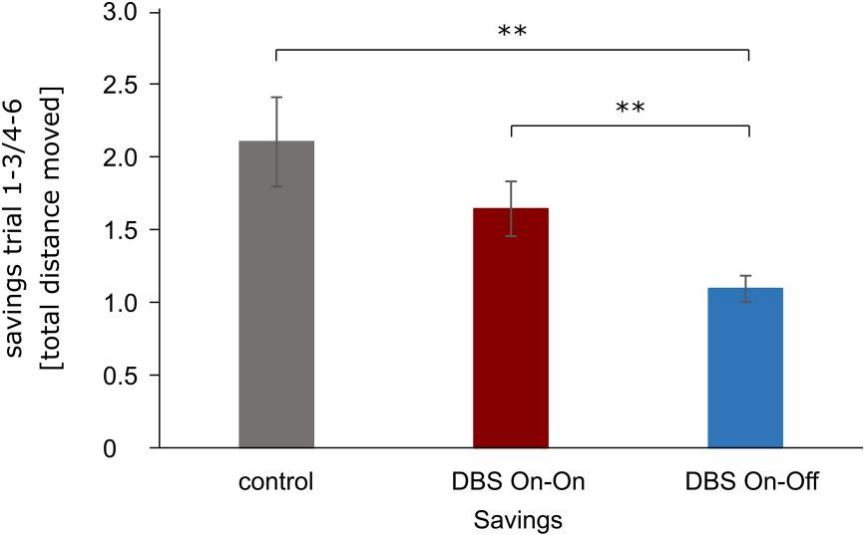
**Savings.** Savings assess the reversal learning performance. Shown are group means of the total distance moved with SEM error bars (Table 4) displaying that the DBS On–Off group performed worse than controls [−1.01, 95% CI (−1.86, −0.16), Cohen’s *d* = −4.3, *P* = 0.02] and the DBS On–On group [−0.55, 95% CI (−1.09, 0.00), Cohen’s *d* = −3.7, *P* = 0.05]. SEM, standard error of the means; DBS, deep brain stimulation.

The quantification of the capacity for spatial reversal learning was determined by means of the measure of ‘savings’ (Fig. 6) which describes the quotient of the path length in trials 1–3 and trials 4–6. An index greater than 1 indicates an improvement of the reversal performance, since the path in the second block was shorter than in the first block. Savings differed statistically significant for the three groups, [Welch’s *F*(2, 21.54) = 7.31, *P* < 0.004]. A Tamhane-T2 showed that healthy controls had a significant higher (*P* = 0.02) saving index than patients with DBS On–Off, [−1.01, 95% CI (−1.86, −0.16), Cohen’s *d* = −4.3]. Also, patients of the DBS On–On group had a higher (*P* = 0.05) saving index than patients with DBS On–Off [−0.55, 95% CI (−1.09, 0.00), Cohen’s *d* = −3.7].

### Quality control

**Figure 7:**
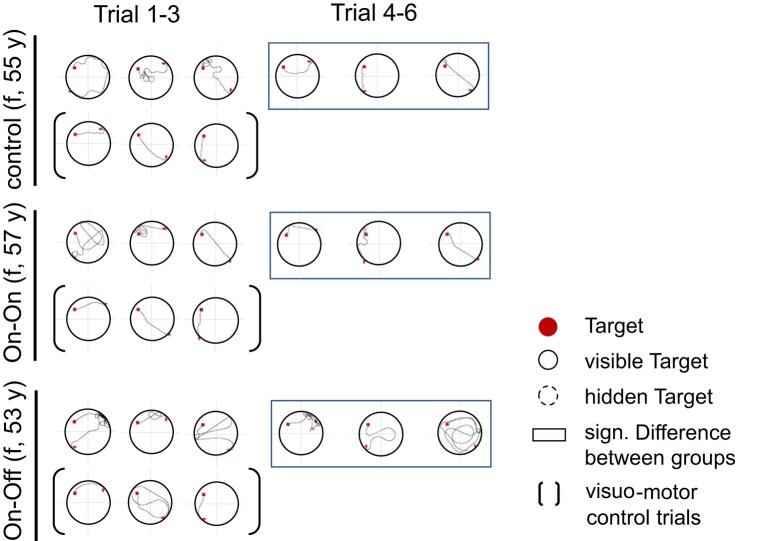
**Navigation paths.** Representative navigation paths from individuals from all three groups (control, On–On and On–Off) during the reversal learning condition with a changed target location into the upper left quadrant. There are longer paths in the trials with changed target location of the patient in the On–Off group. The black frame visualizes significant differences between trials 4 and 6 between groups (see results; Fig. 6 and Tables 3 and 4). sig., significant; f, female.

In the trials 7–9, the box was visible again and the evaluation served as a motor control condition (see also Fig. 7). There were no significant differences in total distance moved [*F*(2, 18.94) = 1.21, *P* = 0.32], which suggests that the effects cannot be explained by motor symptoms in DBS On–Off condition (Table 5).

**Table 5:** Reversal learning

Parameter	DBS On–On	DBS On–Off	ANOVA
*Labeling*			
Learner	0.62 (0.14)	0.54 (0.14)	*χ* ^2^ = 0.62, *P* = 0.70^a^
Reversal Learner	0.46 (0.14)	0.15 (0.10)	*χ* ^2^ = 3.85, *P* = 0.10^a^
Real-Reversal Learner	0.23 (0.12)	0.08 (0.08)	*χ* ^2^ = 12.5, ***P* < 0.001**^a^ ***

*Note*. Data were given as mean relative frequencies (SEM). Significant *P*-values are printed in bold type and are marked with a certain number of stars depending on the degree of significance.

Tests for evaluation were ^a^Kruskal–Wallis test, ANOVA otherwise.

A Kruskal–Wallis did not show any significant differences regarding the classification into ‘Learner’ and ‘Reversal Learner’ between groups of stimulation. Accordingly, in the DBS On–On group, many persons could be classified as ‘Learner’ as in the DBS On–Off group (*χ*^2^ = 0.62, *P* = 0.70). The same applies to ‘Reversal Learner’ (*χ*^2^ = 3.85, *P* = 0.10). Only the difference in the number of real-Reversal Learners was different between patient groups. In the DBS On–On group, there were significantly more real-Reversal Learners than in the DBS On–Off group (*χ*^2^ = 12.5, *P* < 0.0014; Table 5).

### Electrode location

**Figure 8:**
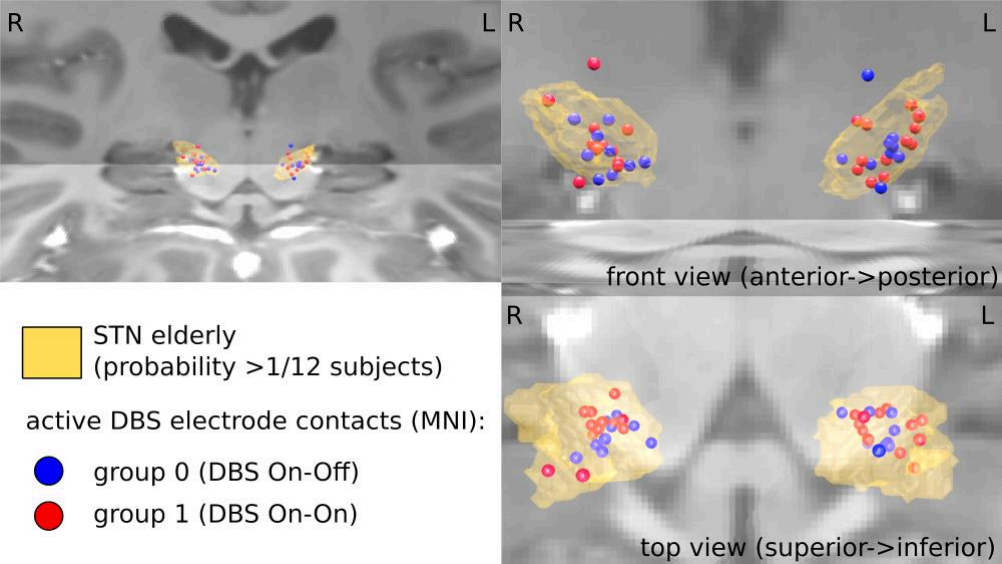
**Electrode location.** Demonstration of the electrode position of DBS in the STN level with 3D STN modelling (yellow formation) from different views. Electrodes are shown as circles separated by groups. Comparable positions of the DBS electrodes are shown for the On–On stimulation group (red circles) compared to the On–Off stimulation group (blue circles). Thus, the positioning of the electrodes does not seem to be able to explain the differences between the spatial performances of the groups. The image was created using the Colin Holmes MNI template.^47^ Image quality was significantly improved after registration, resampling with 0.5 mm^3^ voxels and averaging (*C*: 0.78 mm^3^, *n* = 7; *D*: 1.0 mm^3^, *n* = 20) in a standard space, allowing delineation of fine structures previously obscured by noise. DBS, deep brain stimulation; STN, subthalamic nucleus; MNI, Montreal Neurological Institute (overview and statistics in Table 1).

The applied stimulation parameters (active electrode X, Y, Z), as well as voltage, frequency and Euclidean distance to the STN, were compared inferentially for the left and right hemispheres between the On–On and On–Off stimulation groups. No significant differences were found (Table 1). Figure 8 illustrates the positions of the DBS electrodes within the STN. Comparable positions between the On–On and On–Off stimulation groups are shown.

## Discussion

This study shows a positive effect of STN-DBS on spatial reversal learning, which was apparently unrelated to age, neuropsychological parameters, severity and duration of the disease, motor impairment and electrode position. The effect of STN-DBS was investigated in a randomized control group design by switching off the stimulation in half of the patients after the learning condition. The patients’ data were also compared with a group of healthy controls. Patients with the stimulator switched off were significantly impaired in our spatial reversal learning paradigm, i.e. needed longer walking distances and search time than patients with the stimulator switched on. Patients with stimulator switched on performed significantly better than those with stimulator switched off but less than healthy age-matched controls.

Spatial navigation in humans is the capability of subjects to move directionally in space towards a target or location and using different cue sources such as landmarks.^48^ In humans, it includes manifold neuronal functions ranging from memory, attention and the perception of visual-spatial features as well as direction and distance.^23,24^ Studies have shown different navigational subsystems within the brain. While the hippocampus is considered a brain structure known to play an important role in episodic memory,^49^ the discovery of hippocampal place cells drew attention to its role in spatial memory.^23,24,50-54^ Experiments using the Morris water maze,^34^ among others, showed that certain aspects of navigation such as place memory are particularly sensitive to CA1 hippocampal damage.^35^ Place memory includes navigation to an unfamiliar location from a changing starting position and navigation that requires recall of previously visited locations.^34,48,54,55^

Cortical-basal ganglia circuits including the striatum are thought to support stimulus–response associations and procedural memory and the dorsolateral striatum is considered an important structure for cued navigation learning.^23,24,26^ These cortical-basal ganglia circuits unidirectionally connect specific neocortical areas to striatal subregions, which subsequently project to downstream structures such as the pallidum and substantia nigra, which in turn connect to thalamic nuclei, which in turn project back to the same neocortical sites of origin.^23^ The dorsolateral striatum plays an important role in ‘habitual’ learning and memory and has been hypothesized to implement a reinforcement learning algorithm to select actions to perform given current input.^56^ Thus, the dorsolateral striatum is considered a structure that learns a relationship between a discrete environmental stimulus (cue) and a rewarded response and guides navigation by approaching the cue that predicts a rewarding outcome.^57^

Both the hippocampus and striatum are thought to control navigation using different strategies and in some conditions compete for behavioural control.^23,24,26,28,57^ The differential role of cortical-basal ganglia circuits including the striatum in navigation can be seen in animal models with hippocampal lesions in which the navigation performance was insensitive to hippocampal lesions in the Morris water maze when piloting or response learning was possible.^23^ The hippocampus and dorsolateral striatum are conceptualized together in the multiple memory systems theory as core structures of spatial and stimulus–response (S–R) processing, respectively.^57^ With the help of the paradigm used here, both types of spatial learning can be studied in combination, which can be seen as a clear strength of the test. However, it must be noted that we did not test the interaction between both systems, as both spatial functions were studied sequentially, considering also that both groups learned in the hippocampus-dependent learning condition.

Parkinson’s disease is a progressive chronic neurological disease of the basal ganglia that is associated primarily with motor impairments, including bradykinesia, muscle stiffness, postural instability and rest tremor. But also behavioural, cognitive and vegetative symptoms are major factors in reduced quality of life in the course of the disease and complicate treatment.^1,2^ For example, a study by Ergun *et al*.^18^ showed that impaired navigation during driving in Parkinson’s disease patients was more likely to be associated with impairments in cognitive and visual control functions than with the motor severity of their disease. A commonly reported deficit is the lack of the ability to adapt one’s behaviour to changes in the environment (‘cognitive flexibility’).^17,58,59^ This inflexibility is accompanied by a strong perseverance and an inability to switch internal strategies and is based on an imbalance of direct and indirect loops in the frontal lobe and basal ganglia circuits. Direct striatal neurons are insufficiently activated and indirect striatum neurons are insufficiently inhibited.^5^ This results in an overactivity of the STN.^3,6^ The STN is a small structure with critical importance for decision-making and behavioural control.^7,8^ An important hypothesis suggests a functional division of the STN into sensorimotor (dorsolateral), cognitive (ventromedial) and limbic (orbital and medial) parts with pronounced connectivity to cortical regions.^2,9,60^ Thus, a number of experiments^61,62^ demonstrated the contribution of the STN, particularly the ventral STN through its role in the hyperdirect pathway involving the prefrontal cortex, to conflicting decisions.^8^ In addition, the STN has been shown to be necessary in scenarios that require stopping or pausing responses.^7^ Thus, the STN appears to be closely related to cognitive flexibility, which is a skill to adapt strategies of cognitive processing to new and unexpected conditions in the environment.^58^ In this way, cognitive flexibility is considered an executive function providing the basis for successful reversal learning.^63^ Reversal learning involves reacting to a change in the environment by first ‘unlearning’ or rupturing the original stimulus-reinforcement bond and then obtaining or ‘switching’ to a new one.^64^

These findings are also complemented by the observation that patients with mild to moderate Parkinson’s disease show spatial deficits that are based on the patients’ impairment to generate rules that can be used flexibly in changing environments, especially when proximal cues are removed.^59^ Thus, Parkinson’s disease patients seem to be impaired to perceive changes and reorient randomly in spatial environments.

In this study, we investigated the function of the STN on the cognitive ability of spatial navigation and in particular on spatial reversal learning (switching behavioural response). Although the exact neuromodulatory mechanisms of bilateral STN-DBS are not completely understood, DBS is thought to interfere with basal ganglia’s increased output performance and thereby improve the functions of its target structures in the cortex.^65^

For assessing the navigational performance in Parkinson’s disease, our spatial navigation paradigm, a variation of the Morris water maze, was used. Previous studies have demonstrated a deterioration in spatial learning in patients with Parkinson’s disease, as evidenced by impaired recognition of changes and incidental reorientations in spatial environments.^36,66^ This is explained by the Parkinson’s disease-specific dopamine deficiency syndrome, which is considered a key pathophysiological feature of Parkinson’s disease, and the associated inability to develop complex behavioural strategies.^67^ For example, depletion of dopamine in the striatum has been shown in rodent models to impair the ability to discriminate spatial changes.^68-70^ The loss of these dopamine-producing neurons spreads progressively during the course of the disease and leads to an associated loss of dopaminergic axons in the striatum. Our results indicate that this functional deficit can be improved by STN-DBS. Parkinson’s patients with STN-DBS seem to be able to learn in a similar way as healthy volunteers.

In the immediate recall (no target available), it was measured how much relative time the test persons searched for the chest in the former target quadrant. This recall describes the stability of place memory, which is a hippocampal-dependent navigation function characterized by the flexible use of many environmental markers instead of single local cues in the case of simple cue-response behaviour.^71^ There were no statistically significant differences between the controls and the entire patient group in this initial place learning part of the experiment even when the subjects were subsequently classified into their later stimulation condition (DBS On–On and DBS On–Off); neither in immediate recall, in which all stimulators of the patients were switched on, nor in delayed recall (after 4 h), in which the stimulator was switched off in half of the patients and Parkinson’s disease was thus untreated. This finding supports the assumption that place learning is associated with hippocampal activity. Thus, Parkinson’s patients seem not to have gross impairments in place learning. In the present study, we cannot absolutely exclude hippocampal dysfunction in the patients as recent studies have shown that in the course of cognitive decline in Parkinson’s disease, atrophy of the hippocampus can also be observed. For example, Foo *et al*.^72^ showed significant atrophy with specific reduction in hippocampal subfield volumes in Parkinson’s disease-MCI (mild cognitive impairment). Also, in a recent study by Filippi *et al*.,^73^ the frontotemporoparietal regions, hippocampus and thalamus are shown to be associated with the transition to severe cognitive impairment.^72,74^ However, patients of our study mastered the hippocampus-based learning phase equally well regardless of stimulators being turned on or off and also just as good as the healthy controls. This suggests that hippocampal place learning is comparable in the patient groups and seems to be largely independent of STN stimulation.

After the four-hour break and switching off the stimulator in half of the patients, the variation of the Morris water maze was continued and the treasure chest was reversed into a different quadrant (spatial reversal learning). From now on, only the distance covered to the target was used as a parameter, since time becomes an unsuitable parameter due to the switching off of the stimulators and the associated untreated symptoms of Parkinson’s disease. There was no significant effect of the group in trials 1–3 of the reversal part, suggesting that all subjects started from a comparable basic level and then progressed differently in the next three trials (4th to 6th).

In trials 4–6, patients of the DBS On–Off group significantly covered more distance to the target than patients of the DBS On–On group and healthy controls. Patients in the DBS On–On group, on the other hand, were able to reach the level of healthy controls and covered the same distance to reach the target. Controls and DBS On–On improved visibly from the first block of trials to the second and covered significantly less distance, while patients in the DBS On–Off group covered as much distance to the target in the second block of trials as in the first block. The effect on the reversal measure ‘savings’ was very large (3–4 standard deviations) compared to healthy controls as well as DBS On–On patients. The DBS Off group showed a comparable search behaviour to a recent study characterized by a more or less targeted search, a strategy that lasted longer and produced longer routes.^36,66^ The results suggest that the function of the STN influences the ability of spatial navigation, in particular spatial reversal learning. However, as we studied acute neuromodulatory effects of STN-DBS on spatial learning, we cannot draw conclusions about long-term effects or habituation effects of chronic STN on navigation in Parkinson’s disease.

After analysis, electrode position and motor restrictions could be excluded as possible interfering variables, since the variables did not differ significantly with regard to group.

Our results support the assumption that spatial learning and navigation are supported by various memory systems in the human brain such as the hippocampus-based navigational system and the striatum-cortex-based system. While hippocampal place learning appears to be largely independent of STN stimulation, striatal reversal learning is supported by STN-DBS.

Cognitive abnormalities in Parkinson’s disease are mainly detected in the areas of attention, memory functions and executive functions. Impairments of executive functions are already present in the early course of the disease and are manifested in deficits in the adaptation of habits, cognitive flexibility and the design of internal patterns of action for goal-oriented action.^75,76^ These deficits are typically identified by tests such as the Wisconsin card sorting test (WCST), the Stroop test and tests of phonetic word fluency. But also our spatial water maze paradigm seems to be a suitable method. It is in the nature of successful navigation strategies that those must be flexible to adapt to changing environments. This spatial flexibility can also be assessed after reversal learning and strategy change.^27^ Reversal learning reflects a change in spatial orientation based on the same navigation strategy as initially performed (e.g. place learning → place learning). In contrast, the strategy change is achieved by a change in the navigation strategy (e.g. place learning → response learning). Here, our maze paradigm captures spatial cognitive flexibility and shows a limited flexibility leading to altered reversal learning in Parkinson’s disease patients without STN-DBS. The reason for this could be the influence of the STN on, for example, executive functions, reward learning, cognitive flexibility, set-shifting, attention, impulsivity and perseverative behaviour,^77^ which in turn influence reversal learning. In contrast to the WCST, the maze paradigm also seems to have a greater ecological validity and relevance for everyday life, since navigation to known and changed places is skill needed daily. However, due to the experimental nature, future studies should compare the spatial reversal test with the WCST.

In conclusion, our results suggest that STN-DBS has a positive effect on the reversal learning of changing spatial locations in Parkinson’s patients. In contrast to the patients whose stimulation was switched off after half the task, they were able to adapt their path more quickly to the new target environment. Our results support the assumption that spatial learning and navigation are supported by various memory systems in the human brain such as the hippocampus-based navigational system and the striatum-cortex-based system. While hippocampal place learning appears to be largely independent of STN stimulation, striatal reversal learning is supported by STN-DBS. These findings suggest that STN-DBS restores spatial navigational deficits and indicates that DBS-STN facilitates cognitive flexibility in a spatial context in Parkinson’s disease.

## Data Availability

The data presented in this work are available upon reasonable request.
